# Supplementary material for: Deletion of wfs1 Impairs Oligodendrocyte Precursor Cells Dorsal Distribution and Myelination Through the wfs1-hmgcs1 Axis in Zebrafish
Source: Biology (Basel). 2026 Aug 16;15(16):1402. doi: 10.3390/biology15161402 (PMC13510151; doi:10.3390/biology15161402)
Supplement: Supplementary file 1 [file biology-15-01402-s001.zip › File S1-Transcriptome sequencing data and differentially expressed genes (DEGs) in wfs1-- zebrafish at 5 dpf/2.KEGG/wfs1KOvsWT/GSEA/WT/MOTOR_PROTEINS/MOTOR_PROTEINS(DRE04814).html]

Details for gene set MOTOR\_PROTEINS(DRE04814)[GSEA]

|  || Dataset | deg.sample |
| Phenotype | sample.cls |
| Upregulated in class | WT |
| GeneSet | MOTOR\_PROTEINS(DRE04814) |
| Enrichment Score (ES) | -0.21040146 |
| Normalized Enrichment Score (NES) | -1.099147 |
| Nominal p-value | 0.37349397 |
| FDR q-value | 0.51858205 |
| FWER p-Value | 1.0 |
Table: GSEA Results Summary

  

Fig 1: Enrichment plot: MOTOR\_PROTEINS(DRE04814)      
 Profile of the Running ES Score & Positions of GeneSet Members on the Rank Ordered List

  

| PROBE | DESCRIPTION (from dataset) | GENE SYMBOL | GENE\_TITLE | RANK IN GENE LIST | RANK METRIC SCORE | RUNNING ES | CORE ENRICHMENT || 1 | 57934\_1 | actb1 |  |  | 1 | 4.982 | 0.0285 | No |
| 2 | 101882034 | LOC101882034 |  |  | 73 | 4.809 | 0.0538 | No |
| 3 | 337226\_1 | zgc:153867 |  |  | 121 | 4.641 | 0.0789 | No |
| 4 | 799461\_2 | dnah12 |  |  | 613 | 2.748 | 0.0791 | No |
| 5 | 556023\_1 | dnah2 |  |  | 756 | 2.461 | 0.0887 | No |
| 6 | 677750 | myl2b |  |  | 901 | 2.226 | 0.0969 | No |
| 7 | 798632 | myl6 |  |  | 950 | 2.168 | 0.1078 | No |
| 8 | 474327 | capza1b |  |  | 1384 | 1.736 | 0.1040 | No |
| 9 | 562052\_1 | kif14 |  |  | 1639 | 1.574 | 0.1050 | No |
| 10 | 571487 | si:dkeyp-92c9.3 |  |  | 1723 | 1.523 | 0.1111 | No |
| 11 | 100124599 | smyhc2 |  |  | 1918 | 1.402 | 0.1129 | No |
| 12 | 100037358 | zgc:163073 |  |  | 2005 | 1.366 | 0.1180 | No |
| 13 | 431777 | tuba7l |  |  | 2071 | 1.338 | 0.1236 | No |
| 14 | 373080 | tuba1b |  |  | 2072 | 1.337 | 0.1313 | No |
| 15 | 30429\_1 | mylpfa |  |  | 2177 | 1.294 | 0.1354 | No |
| 16 | 796782 | myo1hb |  |  | 2340 | 1.232 | 0.1373 | No |
| 17 | 565563 | kif5c |  |  | 2416 | 1.202 | 0.1419 | No |
| 18 | 561639 | dnah7 |  |  | 2480 | 1.179 | 0.1466 | No |
| 19 | 100334351 | dnah3 |  |  | 2623 | 1.138 | 0.1486 | No |
| 20 | 558408 | tnni1a |  |  | 2633 | 1.134 | 0.1548 | No |
| 21 | 402809 | tnni2a.3 |  |  | 2666 | 1.122 | 0.1603 | No |
| 22 | novel.875 | - |  |  | 2725 | 1.104 | 0.1647 | No |
| 23 | 563648 | dnai2a |  |  | 3132 | 1.005 | 0.1576 | No |
| 24 | 110439888 | LOC110439888 |  |  | 3177 | 0.998 | 0.1620 | No |
| 25 | 573216 | tuba1a |  |  | 3240 | 0.984 | 0.1656 | No |
| 26 | 555705 | dnai1.2 |  |  | 3417 | 0.949 | 0.1655 | No |
| 27 | 553436 | kif6 |  |  | 3547 | 0.921 | 0.1667 | No |
| 28 | 799461\_1 | dnah12 |  |  | 3796 | 0.869 | 0.1638 | No |
| 29 | 337223 | tuba8l |  |  | 3910 | 0.846 | 0.1651 | No |
| 30 | 570210 | zgc:86709 |  |  | 3984 | 0.834 | 0.1675 | No |
| 31 | 30429 | mylpfa |  |  | 4031 | 0.822 | 0.1708 | No |
| 32 | 407638 | dctn1a |  |  | 4111 | 0.809 | 0.1729 | No |
| 33 | 556141 | myo7ab |  |  | 4282 | 0.779 | 0.1720 | No |
| 34 | 559562 | si:ch211-94l19.4 |  |  | 4479 | 0.748 | 0.1701 | No |
| 35 | 569779 | myo10 |  |  | 4915 | 0.691 | 0.1603 | No |
| 36 | 560956 | myo9b |  |  | 5109 | 0.666 | 0.1580 | No |
| 37 | 557863 | kif17 |  |  | 5486 | 0.635 | 0.1497 | No |
| 38 | novel.441 | - |  |  | 5583 | 0.628 | 0.1503 | No |
| 39 | 402901 | dynlt3 |  |  | 5666 | 0.620 | 0.1512 | No |
| 40 | 550267 | kif3a |  |  | 5673 | 0.620 | 0.1546 | No |
| 41 | 567045 | kif16bb |  |  | 6005 | 0.585 | 0.1474 | No |
| 42 | 108181090 | LOC108181090 |  |  | 6212 | 0.567 | 0.1442 | No |
| 43 | 436779 | lyrm1 |  |  | 6221 | 0.566 | 0.1472 | No |
| 44 | 561788 | kif22 |  |  | 6397 | 0.547 | 0.1447 | No |
| 45 | 798868 | LOC798868 |  |  | 6449 | 0.542 | 0.1462 | No |
| 46 | 568760 | si:dkey-110c1.10 |  |  | 6469 | 0.540 | 0.1487 | No |
| 47 | 570210\_2 | zgc:86709 |  |  | 6553 | 0.532 | 0.1491 | No |
| 48 | 393452 | dync2li1 |  |  | 6621 | 0.526 | 0.1500 | No |
| 49 | 550267\_2 | kif3a |  |  | 6669 | 0.522 | 0.1515 | No |
| 50 | 110439139 | LOC110439139 |  |  | 6671 | 0.522 | 0.1545 | No |
| 51 | 553797 | wdr78 |  |  | 6675 | 0.522 | 0.1574 | No |
| 52 | 567402 | myo5b |  |  | 6871 | 0.503 | 0.1541 | No |
| 53 | 792024 | si:ch73-199e17.1 |  |  | 7117 | 0.481 | 0.1491 | No |
| 54 | 393154 | tuba8l4 |  |  | 7289 | 0.467 | 0.1464 | No |
| 55 | 103909932 | LOC103909932 |  |  | 7464 | 0.456 | 0.1435 | No |
| 56 | 100136875 | kif2c |  |  | 7538 | 0.452 | 0.1437 | No |
| 57 | 100007502 | kif20bb |  |  | 8005 | 0.423 | 0.1314 | No |
| 58 | 560592 | myo15aa |  |  | 8479 | 0.404 | 0.1187 | No |
| 59 | 795537\_1 | myo15b |  |  | 8529 | 0.401 | 0.1195 | No |
| 60 | 555290 | myo10l3 |  |  | 8539 | 0.400 | 0.1215 | No |
| 61 | 322509 | acta2 |  |  | 8570 | 0.398 | 0.1228 | No |
| 62 | 572690 | dynll2b |  |  | 8704 | 0.388 | 0.1208 | No |
| 63 | 336165 | myl1 |  |  | 8762 | 0.384 | 0.1212 | No |
| 64 | 101884886 | si:ch211-235m3.5 |  |  | 8931 | 0.372 | 0.1180 | No |
| 65 | novel.442 | - |  |  | 9127 | 0.358 | 0.1139 | No |
| 66 | 100329748\_1 | LOC100329748 |  |  | 9141 | 0.357 | 0.1156 | No |
| 67 | 793121\_1 | si:ch211-257p13.3 |  |  | 9211 | 0.352 | 0.1154 | No |
| 68 | 100149555 | stard9 |  |  | 9401 | 0.340 | 0.1114 | No |
| 69 | 393209 | kif18a |  |  | 9732 | 0.319 | 0.1027 | No |
| 70 | 393664 | tuba8l2 |  |  | 9767 | 0.318 | 0.1035 | No |
| 71 | 108190403\_3 | LOC108190403 |  |  | 9788 | 0.316 | 0.1047 | No |
| 72 | 373083\_1 | dynll2a |  |  | 9884 | 0.311 | 0.1034 | No |
| 73 | 565345 | myo1f |  |  | 9987 | 0.304 | 0.1019 | No |
| 74 | 327377 | tuba4l |  |  | 9990 | 0.304 | 0.1036 | No |
| 75 | 566034 | myo7bb |  |  | 10221 | 0.290 | 0.0980 | No |
| 76 | 571520 | kif13ba |  |  | 10256 | 0.287 | 0.0986 | No |
| 77 | 641421 | tubb2b |  |  | 10342 | 0.282 | 0.0975 | No |
| 78 | novel.445 | - |  |  | 10371 | 0.281 | 0.0982 | No |
| 79 | novel.440 | - |  |  | 10457 | 0.275 | 0.0971 | No |
| 80 | 100537719 | bicdl1 |  |  | 10461 | 0.275 | 0.0986 | No |
| 81 | 562279 | myo19 |  |  | 10493 | 0.273 | 0.0992 | No |
| 82 | novel.233 | - |  |  | 10536 | 0.270 | 0.0994 | No |
| 83 | 100333558 | kif3c |  |  | 10805 | 0.255 | 0.0923 | No |
| 84 | 100332329 | LOC100332329 |  |  | 10901 | 0.249 | 0.0908 | No |
| 85 | 406811\_2 | zgc:55461 |  |  | 11028 | 0.242 | 0.0882 | No |
| 86 | 415183\_1 | tubd1 |  |  | 11046 | 0.241 | 0.0890 | No |
| 87 | 100317281 | kif13bb |  |  | 11180 | 0.234 | 0.0861 | No |
| 88 | 393139 | actr10 |  |  | 11498 | 0.217 | 0.0773 | No |
| 89 | 567643 | kif26aa |  |  | 11658 | 0.208 | 0.0735 | No |
| 90 | 573988 | kif15 |  |  | 11732 | 0.204 | 0.0724 | No |
| 91 | 100537244 | myl2a |  |  | 11772 | 0.202 | 0.0723 | No |
| 92 | 386701 | tubb5 |  |  | 11807 | 0.201 | 0.0724 | No |
| 93 | 100005529\_2 | dnal4b |  |  | 11815 | 0.200 | 0.0733 | No |
| 94 | 436902\_1 | tnni1d |  |  | 12009 | 0.191 | 0.0683 | No |
| 95 | 565913 | kif19 |  |  | 12140 | 0.183 | 0.0652 | No |
| 96 | 544651 | kif7 |  |  | 12202 | 0.179 | 0.0643 | No |
| 97 | 767729 | dync1i2a |  |  | 12259 | 0.176 | 0.0635 | No |
| 98 | 325449 | kif20a |  |  | 12435 | 0.167 | 0.0589 | No |
| 99 | 559005 | myo18ab |  |  | 12648 | 0.157 | 0.0531 | No |
| 100 | 445048 | dnal1 |  |  | 12822 | 0.149 | 0.0485 | No |
| 101 | 110438357 | LOC110438357 |  |  | 12866 | 0.147 | 0.0480 | No |
| 102 | 101884977 | dnai2b |  |  | 12948 | 0.143 | 0.0462 | No |
| 103 | 541515 | myo1b |  |  | 13013 | 0.139 | 0.0450 | No |
| 104 | 100148193\_1 | dnah6 |  |  | 13042 | 0.138 | 0.0449 | No |
| 105 | 30627 | kif23 |  |  | 13079 | 0.136 | 0.0446 | No |
| 106 | 336681 | tubb4b |  |  | 13139 | 0.133 | 0.0434 | No |
| 107 | 445209\_1 | dnal4a |  |  | 13627 | 0.110 | 0.0287 | No |
| 108 | 393796 | kif4 |  |  | 13660 | 0.108 | 0.0283 | No |
| 109 | 556606 | myo1ha |  |  | 13700 | 0.107 | 0.0276 | No |
| 110 | 335083 | capzb |  |  | 13723 | 0.106 | 0.0275 | No |
| 111 | 795537 | myo15b |  |  | 13749 | 0.104 | 0.0274 | No |
| 112 | 100333131 | wdr63 |  |  | 13952 | 0.095 | 0.0215 | No |
| 113 | 195818 | kif11 |  |  | 13963 | 0.094 | 0.0217 | No |
| 114 | 562188\_1 | myo5aa |  |  | 13981 | 0.094 | 0.0217 | No |
| 115 | 100001191\_1 | si:ch211-147k9.8 |  |  | 14223 | 0.081 | 0.0146 | No |
| 116 | 573266\_1 | dync1li2 |  |  | 14230 | 0.081 | 0.0148 | No |
| 117 | 402814 | myo3a |  |  | 14293 | 0.078 | 0.0133 | No |
| 118 | 445029 | tnni2b.2 |  |  | 14527 | 0.068 | 0.0063 | No |
| 119 | 553436\_1 | kif6 |  |  | 14664 | 0.062 | 0.0024 | No |
| 120 | 555285 | myo1ea |  |  | 14720 | 0.059 | 0.0010 | No |
| 121 | 30453 | kifc1 |  |  | 14864 | 0.052 | -0.0032 | No |
| 122 | 555454 | myh10 |  |  | 14890 | 0.051 | -0.0037 | No |
| 123 | 568760\_1 | si:dkey-110c1.10 |  |  | 15147 | 0.039 | -0.0116 | No |
| 124 | 406297 | dynll1 |  |  | 15150 | 0.039 | -0.0115 | No |
| 125 | 567536 | myo1ca |  |  | 15225 | 0.036 | -0.0136 | No |
| 126 | 100317899 | dnah1 |  |  | 15400 | 0.027 | -0.0190 | No |
| 127 | 100331282 | kif13a |  |  | 15586 | 0.018 | -0.0247 | No |
| 128 | 445164 | tuba8l3 |  |  | 15740 | 0.009 | -0.0295 | No |
| 129 | 777640 | zgc:153929 |  |  | 15936 | 0.001 | -0.0357 | No |
| 130 | 799274 | kif5ab |  |  | 16053 | -0.003 | -0.0393 | No |
| 131 | 334108 | kif5bb |  |  | 16350 | -0.012 | -0.0486 | No |
| 132 | 100329813\_1 | LOC100329813 |  |  | 16377 | -0.013 | -0.0494 | No |
| 133 | 58004 | kif26ab |  |  | 16385 | -0.014 | -0.0495 | No |
| 134 | 394163 | dynlrb1 |  |  | 16413 | -0.015 | -0.0503 | No |
| 135 | novel.439 | - |  |  | 16475 | -0.017 | -0.0521 | No |
| 136 | 393882 | tubg1 |  |  | 16476 | -0.017 | -0.0520 | No |
| 137 | 100333605 | kif26bb |  |  | 16503 | -0.017 | -0.0527 | No |
| 138 | 100329813 | LOC100329813 |  |  | 16620 | -0.021 | -0.0563 | No |
| 139 | 58082 | tnnc2 |  |  | 16621 | -0.021 | -0.0562 | No |
| 140 | 100332402 | dync2h1 |  |  | 16726 | -0.023 | -0.0593 | No |
| 141 | 321552 | smyhc1 |  |  | 16751 | -0.024 | -0.0600 | No |
| 142 | 562676 | myo5c |  |  | 16756 | -0.024 | -0.0599 | No |
| 143 | 566459 | myo1cb |  |  | 17090 | -0.036 | -0.0703 | No |
| 144 | 570502 | kif21b |  |  | 17243 | -0.043 | -0.0749 | No |
| 145 | 560945 | myh7bb |  |  | 17419 | -0.050 | -0.0801 | No |
| 146 | 393960 | tube1 |  |  | 17552 | -0.056 | -0.0840 | No |
| 147 | 445472 | myo6b |  |  | 17671 | -0.061 | -0.0874 | No |
| 148 | 566880 | myo18aa |  |  | 17693 | -0.062 | -0.0877 | No |
| 149 | 558219 | kif25 |  |  | 17755 | -0.065 | -0.0892 | No |
| 150 | 100002038\_1 | si:rp71-17i16.4 |  |  | 17829 | -0.069 | -0.0911 | No |
| 151 | 562052 | kif14 |  |  | 17890 | -0.071 | -0.0926 | No |
| 152 | 100330443 | kif1aa |  |  | 17900 | -0.072 | -0.0925 | No |
| 153 | 100535326 | dnah5 |  |  | 17997 | -0.075 | -0.0951 | No |
| 154 | 100006612 | myo9aa |  |  | 18099 | -0.081 | -0.0978 | No |
| 155 | 108190403 | LOC108190403 |  |  | 18184 | -0.084 | -0.1000 | No |
| 156 | 100008376 | wu:fd14a01 |  |  | 18193 | -0.085 | -0.0998 | No |
| 157 | 569545 | kif26ba |  |  | 18292 | -0.089 | -0.1024 | No |
| 158 | 570113 | myo15ab |  |  | 18293 | -0.089 | -0.1019 | No |
| 159 | 108190403\_2 | LOC108190403 |  |  | 18370 | -0.092 | -0.1038 | No |
| 160 | 568367 | kifc3 |  |  | 18456 | -0.096 | -0.1059 | No |
| 161 | 550458 | tubb6 |  |  | 18472 | -0.097 | -0.1058 | No |
| 162 | 450006 | myl9a |  |  | 18511 | -0.099 | -0.1064 | No |
| 163 | 561431 | myh9b |  |  | 18683 | -0.107 | -0.1112 | No |
| 164 | 492493 | tnni2a.1 |  |  | 18945 | -0.120 | -0.1188 | No |
| 165 | 393609\_1 | myo1eb |  |  | 18961 | -0.120 | -0.1186 | No |
| 166 | 100148193 | dnah6 |  |  | 18980 | -0.120 | -0.1185 | No |
| 167 | 406681 | actr1 |  |  | 19140 | -0.128 | -0.1228 | No |
| 168 | 321552\_1 | smyhc1 |  |  | 19284 | -0.135 | -0.1265 | No |
| 169 | 326719 | myl12.1 |  |  | 19292 | -0.135 | -0.1260 | No |
| 170 | 554168 | myh11a |  |  | 19328 | -0.137 | -0.1263 | No |
| 171 | 335499 | klc1b |  |  | 19335 | -0.137 | -0.1257 | No |
| 172 | 353248 | tnnt2c |  |  | 19676 | -0.151 | -0.1356 | No |
| 173 | 447884 | tctex1d1 |  |  | 19788 | -0.156 | -0.1382 | No |
| 174 | 100331282\_1 | kif13a |  |  | 19800 | -0.157 | -0.1377 | No |
| 175 | 100101641 | kif3b |  |  | 19833 | -0.158 | -0.1378 | No |
| 176 | 333938 | myh9a |  |  | 20081 | -0.170 | -0.1446 | No |
| 177 | 570771 | kif1c |  |  | 20138 | -0.172 | -0.1454 | No |
| 178 | 445473 | myo6a |  |  | 20344 | -0.181 | -0.1509 | No |
| 179 | 554127 | tubb2 |  |  | 20659 | -0.196 | -0.1597 | No |
| 180 | 337084 | myl12.2 |  |  | 20670 | -0.197 | -0.1589 | No |
| 181 | 327029 | capza1a |  |  | 20750 | -0.201 | -0.1602 | No |
| 182 | 252846 | myo7aa |  |  | 20860 | -0.208 | -0.1625 | No |
| 183 | 415164 | zgc:86725 |  |  | 20902 | -0.209 | -0.1626 | No |
| 184 | 751665 | tnni1c |  |  | 20952 | -0.213 | -0.1629 | No |
| 185 | 449979\_1 | kif20ba |  |  | 21048 | -0.218 | -0.1647 | No |
| 186 | 335110 | dync1h1 |  |  | 21161 | -0.224 | -0.1670 | No |
| 187 | 100002040 | myhb |  |  | 21203 | -0.226 | -0.1670 | No |
| 188 | 567790 | LOC567790 |  |  | 21265 | -0.228 | -0.1676 | No |
| 189 | 335798 | zgc:65894 |  |  | 21348 | -0.234 | -0.1688 | No |
| 190 | 431767 | dctn3 |  |  | 21436 | -0.238 | -0.1702 | No |
| 191 | 100005529\_1 | dnal4b |  |  | 21438 | -0.238 | -0.1689 | No |
| 192 | 436770 | dctn5 |  |  | 21459 | -0.238 | -0.1682 | No |
| 193 | 100535869 | LOC100535869 |  |  | 21662 | -0.249 | -0.1731 | No |
| 194 | 406437 | tpm4a |  |  | 21677 | -0.250 | -0.1722 | No |
| 195 | 474327\_2 | capza1b |  |  | 21678 | -0.250 | -0.1707 | No |
| 196 | 793976 | tctex1d4 |  |  | 21722 | -0.252 | -0.1706 | No |
| 197 | 447906 | kifap3a |  |  | 21844 | -0.259 | -0.1730 | No |
| 198 | 767754 | cenpe |  |  | 21964 | -0.265 | -0.1752 | No |
| 199 | 574000 | tnnt2d |  |  | 22059 | -0.270 | -0.1767 | No |
| 200 | 562365 | klc2 |  |  | 22099 | -0.273 | -0.1763 | No |
| 201 | 406303 | tuba2 |  |  | 22113 | -0.274 | -0.1752 | No |
| 202 | 324244 | tpm4b |  |  | 22140 | -0.275 | -0.1744 | No |
| 203 | 563752 | myo16 |  |  | 22245 | -0.281 | -0.1761 | No |
| 204 | 566481 | kif21a |  |  | 22339 | -0.286 | -0.1774 | No |
| 205 | 565267 | myo5ab |  |  | 22447 | -0.293 | -0.1791 | No |
| 206 | 569394 | klc3 |  |  | 22448 | -0.293 | -0.1775 | No |
| 207 | 393609 | myo1eb |  |  | 22461 | -0.293 | -0.1762 | No |
| 208 | 797363 | si:dkey-26i13.8 |  |  | 22601 | -0.302 | -0.1788 | No |
| 209 | 408256 | actc1a |  |  | 22616 | -0.303 | -0.1775 | No |
| 210 | 394068 | tnni2a.2 |  |  | 22705 | -0.308 | -0.1786 | No |
| 211 | 100334507 | kif16ba |  |  | 22713 | -0.308 | -0.1770 | No |
| 212 | 563459 | kif5ba |  |  | 22998 | -0.327 | -0.1841 | No |
| 213 | 406493 | myl9b |  |  | 23170 | -0.339 | -0.1876 | No |
| 214 | 561525 | dync1i1 |  |  | 23257 | -0.345 | -0.1884 | No |
| 215 | 552915 | dnah9l |  |  | 23297 | -0.347 | -0.1876 | No |
| 216 | novel.934 | - |  |  | 23418 | -0.356 | -0.1894 | No |
| 217 | 550479 | dctn4 |  |  | 23495 | -0.360 | -0.1897 | No |
| 218 | 795591 | si:ch211-114n24.6 |  |  | 23557 | -0.364 | -0.1896 | No |
| 219 | 58082\_1 | tnnc2 |  |  | 23591 | -0.366 | -0.1885 | No |
| 220 | 415191 | tnni1al |  |  | 23592 | -0.367 | -0.1864 | No |
| 221 | 566086 | kif5aa |  |  | 23616 | -0.368 | -0.1850 | No |
| 222 | 564573 | myo3b |  |  | 23735 | -0.376 | -0.1866 | No |
| 223 | 100884147 | myh7ba |  |  | 23742 | -0.376 | -0.1846 | No |
| 224 | 415209 | tpm2 |  |  | 23895 | -0.386 | -0.1873 | No |
| 225 | 556703\_1 | tnnt2e |  |  | 23899 | -0.386 | -0.1851 | No |
| 226 | 100003026 | dctn1b |  |  | 24106 | -0.400 | -0.1894 | No |
| 227 | 266676 | dnah9 |  |  | 24155 | -0.404 | -0.1886 | No |
| 228 | 407658 | acta1b |  |  | 24188 | -0.406 | -0.1873 | No |
| 229 | 394141 | dctn2 |  |  | 24688 | -0.429 | -0.2006 | No |
| 230 | 100333835 | LOC100333835 |  |  | 24698 | -0.430 | -0.1984 | No |
| 231 | 447926 | zgc:103670 |  |  | 24706 | -0.431 | -0.1962 | No |
| 232 | 100148193\_2 | dnah6 |  |  | 24974 | -0.444 | -0.2021 | No |
| 233 | 566766 | kif1ab |  |  | 24977 | -0.444 | -0.1996 | No |
| 234 | 550569 | myl10 |  |  | 24980 | -0.444 | -0.1971 | No |
| 235 | 30616 | vmhc |  |  | 25400 | -0.475 | -0.2077 | Yes |
| 236 | 574004 | myl4 |  |  | 25467 | -0.481 | -0.2070 | Yes |
| 237 | 57935 | actb2 |  |  | 25530 | -0.487 | -0.2062 | Yes |
| 238 | 550547 | kif3cb |  |  | 25537 | -0.488 | -0.2036 | Yes |
| 239 | 100003572 | tnnt3b |  |  | 25562 | -0.490 | -0.2015 | Yes |
| 240 | 386711 | myh6 |  |  | 25613 | -0.495 | -0.2003 | Yes |
| 241 | 393928 | klc1a |  |  | 25735 | -0.506 | -0.2012 | Yes |
| 242 | 562188 | myo5aa |  |  | 25834 | -0.513 | -0.2014 | Yes |
| 243 | 100331199 | tnnt2b |  |  | 25902 | -0.520 | -0.2005 | Yes |
| 244 | 335340 | dnah5l |  |  | 25991 | -0.528 | -0.2003 | Yes |
| 245 | 108190403\_1 | LOC108190403 |  |  | 26196 | -0.547 | -0.2036 | Yes |
| 246 | 246275 | myhz2 |  |  | 26211 | -0.549 | -0.2009 | Yes |
| 247 | 406257 | zgc:56231 |  |  | 26314 | -0.558 | -0.2010 | Yes |
| 248 | 100536916 | hook3 |  |  | 26321 | -0.558 | -0.1979 | Yes |
| 249 | novel.446 | - |  |  | 26406 | -0.567 | -0.1974 | Yes |
| 250 | 556103 | dync1li1 |  |  | 26421 | -0.569 | -0.1945 | Yes |
| 251 | 373083 | dynll2a |  |  | 26592 | -0.587 | -0.1966 | Yes |
| 252 | 566674 | myo10l1 |  |  | 26631 | -0.591 | -0.1944 | Yes |
| 253 | 567632 | dnali1 |  |  | 26639 | -0.592 | -0.1912 | Yes |
| 254 | 337770 | si:ch73-187m15.4 |  |  | 26661 | -0.594 | -0.1885 | Yes |
| 255 | 558627 | tnnt1 |  |  | 26703 | -0.599 | -0.1863 | Yes |
| 256 | 58114 | actc1b |  |  | 26722 | -0.601 | -0.1835 | Yes |
| 257 | 556141\_1 | myo7ab |  |  | 27027 | -0.632 | -0.1895 | Yes |
| 258 | 449825 | zgc:101560 |  |  | 27039 | -0.633 | -0.1862 | Yes |
| 259 | 447930 | mylpfb |  |  | 27171 | -0.644 | -0.1867 | Yes |
| 260 | novel.444 | - |  |  | 27309 | -0.650 | -0.1873 | Yes |
| 261 | 58142 | myhz1.1 |  |  | 27390 | -0.657 | -0.1861 | Yes |
| 262 | 799300 | myhz1.2 |  |  | 27482 | -0.671 | -0.1851 | Yes |
| 263 | 103911411\_1 | LOC103911411 |  |  | 27699 | -0.701 | -0.1879 | Yes |
| 264 | 768300 | vmhcl |  |  | 27872 | -0.725 | -0.1892 | Yes |
| 265 | novel.443 | - |  |  | 27883 | -0.726 | -0.1854 | Yes |
| 266 | 100329748 | LOC100329748 |  |  | 27955 | -0.737 | -0.1834 | Yes |
| 267 | 494164 | tnni2a.4 |  |  | 28066 | -0.751 | -0.1826 | Yes |
| 268 | 558552 | tubb1 |  |  | 28296 | -0.785 | -0.1853 | Yes |
| 269 | novel.447 | - |  |  | 28302 | -0.786 | -0.1810 | Yes |
| 270 | 553438 | si:dkey-86l18.8 |  |  | 28323 | -0.790 | -0.1771 | Yes |
| 271 | 415164\_1 | zgc:86725 |  |  | 28382 | -0.801 | -0.1744 | Yes |
| 272 | 100001191 | si:ch211-147k9.8 |  |  | 28494 | -0.818 | -0.1732 | Yes |
| 273 | 570210\_1 | zgc:86709 |  |  | 28539 | -0.828 | -0.1698 | Yes |
| 274 | 565740 | dynlt1 |  |  | 28568 | -0.834 | -0.1659 | Yes |
| 275 | 415175 | tnnc1b |  |  | 28699 | -0.858 | -0.1651 | Yes |
| 276 | 767746 | zgc:153426 |  |  | 28740 | -0.866 | -0.1615 | Yes |
| 277 | 556703 | tnnt2e |  |  | 28776 | -0.874 | -0.1576 | Yes |
| 278 | 369190 | kif1b |  |  | 28777 | -0.874 | -0.1526 | Yes |
| 279 | 550249 | tnni2b.1 |  |  | 29028 | -0.927 | -0.1552 | Yes |
| 280 | 58071 | tnnt2a |  |  | 29115 | -0.946 | -0.1525 | Yes |
| 281 | 561612 | dync1i2b |  |  | 29202 | -0.974 | -0.1496 | Yes |
| 282 | 573122 | tuba1c |  |  | 29368 | -1.020 | -0.1490 | Yes |
| 283 | 563091 | si:dkey-206m15.8 |  |  | 29402 | -1.032 | -0.1441 | Yes |
| 284 | 30592 | myl7 |  |  | 29608 | -1.098 | -0.1443 | Yes |
| 285 | 100124599\_1 | smyhc2 |  |  | 29618 | -1.101 | -0.1383 | Yes |
| 286 | 64671 | cmlc1 |  |  | 29619 | -1.102 | -0.1320 | Yes |
| 287 | 58084 | tnnt3a |  |  | 29868 | -1.191 | -0.1330 | Yes |
| 288 | 553694 | zgc:112242 |  |  | 29882 | -1.195 | -0.1266 | Yes |
| 289 | 569992 | si:dkeyp-86b9.1 |  |  | 29914 | -1.207 | -0.1207 | Yes |
| 290 | 569779\_1 | myo10 |  |  | 30057 | -1.267 | -0.1179 | Yes |
| 291 | 799461 | dnah12 |  |  | 30092 | -1.282 | -0.1117 | Yes |
| 292 | 100008070 | ns:zf-e68 |  |  | 30102 | -1.286 | -0.1046 | Yes |
| 293 | 798632\_1 | myl6 |  |  | 30126 | -1.302 | -0.0978 | Yes |
| 294 | 494070 | tnni1b |  |  | 30376 | -1.434 | -0.0975 | Yes |
| 295 | 550445 | acta1a |  |  | 30411 | -1.455 | -0.0903 | Yes |
| 296 | 100037358\_1 | zgc:163073 |  |  | 30530 | -1.526 | -0.0853 | Yes |
| 297 | 556023 | dnah2 |  |  | 30558 | -1.550 | -0.0772 | Yes |
| 298 | 353247 | tnnc1a |  |  | 30580 | -1.568 | -0.0689 | Yes |
| 299 | 100149148 | myha |  |  | 30777 | -1.740 | -0.0652 | Yes |
| 300 | 406811\_1 | zgc:55461 |  |  | 30849 | -1.809 | -0.0570 | Yes |
| 301 | 57934 | actb1 |  |  | 31071 | -2.048 | -0.0523 | Yes |
| 302 | 334274 | myhc4 |  |  | 31301 | -2.432 | -0.0456 | Yes |
| 303 | 30429\_2 | mylpfa |  |  | 31431 | -2.730 | -0.0341 | Yes |
| 304 | 337226 | zgc:153867 |  |  | 31724 | -3.881 | -0.0211 | Yes |
| 305 | 474327\_1 | capza1b |  |  | 31799 | -4.600 | 0.0029 | Yes |
Table: GSEA details [plain text format]

  

Fig 2: MOTOR\_PROTEINS(DRE04814)      
 Blue-Pink O' Gram in the Space of the Analyzed GeneSet

  

Fig 3: MOTOR\_PROTEINS(DRE04814): Random ES distribution      
 Gene set null distribution of ES for **MOTOR\_PROTEINS(DRE04814)**
